# Sugar Maple Litter Decay Rates Are Reduced More Strongly by Drought Than by American Beech Proliferation in the Understory

**DOI:** 10.1002/ece3.71416

**Published:** 2025-05-19

**Authors:** William F. J. Parsons, Claudele Ghotsa Mekontchou, Audrey Maheu, David Rivest

**Affiliations:** ^1^ Département de Biologie Université de Sherbrooke Sherbrooke Quebec Canada; ^2^ Département des sciences naturelles & Institut des Sciences de la forêt tempérée (ISFORT) Université du Québec en Outaouais Ripon Quebec Canada

**Keywords:** *Acer saccharum*, bag mesh size, decomposition, *Fagus grandifolia*, leaf mixtures, rainfall exclusion

## Abstract

Recurrent drought threatens high‐latitude deciduous forests in eastern North America. The decline of sugar maple ( *Acer saccharum* ) at its northern limits under additional stress imposed by changing climate regimes and its replacement by American beech ( *Fagus grandifolia* ) cannot maintain the ecosystem services that the former provides, including its nutrient‐rich leaf litter. In 2022, we incubated litter bags in three maple stands (Kenauk Reserve, Quebec) where beech saplings proliferated (Proliferation = Yes) versus three beech‐free ones (Proliferation = No), in which paired plots were established with rainfall excluders versus rainfall accessibility in summer 2021 and 2022. Moisture was two‐fold higher in rainfall‐accessible soils (Rainfall exclusion, No), but half as spatially variable (CV%) as excluder plot soils (Exclusion, Yes). Mesh bags (mm apertures: 50, Large; 20, Medium; 0.1, Small) that were filled with maple or beech leaves, or their 50:50 mixtures (Species), were deployed in June 2022 within the 12 plots, with 30‐, 60‐, and 90‐day removals. Mass loss did not change with mesh size in a consistent manner over 90 days (initial prediction: L > M > S). We estimated *k*‐values (year^−1^) by extending the linearized exponential decay model to 12 Proliferation‐Exclusion‐Species combinations. Maple decayed 16%–30% faster than beech when soil moisture was not limited. Mass loss rates were 2.7–4.13 (beech saplings, Yes) and 2.3–2.9 (beech, No) times higher under rainfall than under moisture deficiency, and ordered: maple ≥ mixed > beech. Separate *k*‐values for mixed‐bag species were higher than their monospecific counterparts, suggesting synergistic behavior. Early leaching is drought‐sensitive; mass losses were 2.5–2.9 times higher under accessible rainfall versus rainfall exclusion. Furthermore, moisture and maple decay may be increased under slowly decaying beech “mulch.” Indeed, slightly higher maple loss rates were observed beneath beech understories, despite possible negative effects of leaf and litter leachates produced by the latter. Recurring drought and shifting stand composition through continued beech‐maple competition may threaten the persistence and productivity of northern hardwood communities, thereby affecting ecosystem functioning, including decomposition and associated biogeochemical transformations.

## Graphical abstract

Tree litter decay rates were estimated for 36 species (3) × rainfall exclusion (2) × American beech proliferation (2) × bag mesh size (3) treatment combinations. Rainfall exclusion dominated effects on instantaneous litter mass loss rates. Beech proliferation in the understory and bag mesh‐size differences exerted much more subtle effects.

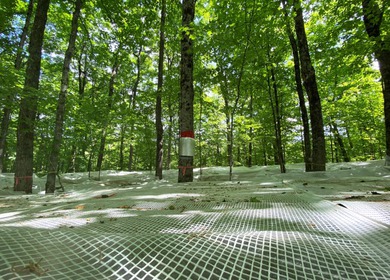

## 1 Introduction

Sugar maple ( *Acer saccharum* Marsh.) is an ecologically, economically, and culturally important tree species dominating canopies of temperate hardwood forests in eastern North America (Godman et al. 1990). The species is a strong competitor for space, light, and soil resources (water, nutrients), particularly in three characteristic bioclimatic domains at its northern range limits in Quebec. Sugar maple typically shares the forest canopy with bitternut hickory ( *Carya cordiformis* [Wangenh.] [K. Koch), American basswood ( *Tilia americana* L.), and yellow birch ( *Betula alleghaniensis* Britt.). While sugar maple can maintain its position in the canopy with understory conditions favoring a diverse vascular plant ground cover and a broad assemblage of advance regeneration (Soubeyrand et al. 2024), decreasing diversity trends and maple dominance have continued since European settlement and land‐use change began in the northern hardwood forests (Gravel et al. 2011; Henry and Walters 2023). In terms of stand composition, gradual replacement of sugar maple by American beech ( *Fagus grandifolia* Ehrh.) as the canopy dominant has been noted recently in northern hardwood forests (Bose et al. 2017b).

American beech and sugar maple share life history traits, accounting for their apparent canopy co‐dominance: slow to moderate growth rates; extremely high shade tolerance; and mast seed production, which often coincides between the two species (Godman et al. 1990; Tubbs and Houston 1990). Beech has less exacting growth requirements than sugar maple, subsequently performing well across a broader range of soil moisture conditions relative to the latter species. Furthermore, chronic sub‐optimal growing conditions (higher temperatures, lower soil moisture, lower soil fertility) for sugar maple that have arisen from stressors and climate effects result in its well‐known widespread decline and death, while apparently leaving beech unaffected (Boakye et al. 2023) and facilitating its proliferation. Beech can out‐perform maple growth (especially at the sapling stage) through greater radial expansion rates of its stems, frequently replacing maple in the understory and sub‐canopy stages despite the imposition of silvicultural interventions to lower its densities (Leduc et al. 2024; see Nelson and Wagner 2014; St‐Jean et al. 2021).

Canopy recruitment of maple is least favorable when its own saplings are present in high densities and experiencing intense intra‐specific competition, or as previously mentioned, when the understory consists of dense beech sapling thickets, where ramet densities can exceed 1500 stems ha^−1^ (Gravel et al. 2011; Bose et al. 2017a). Dense thickets of beech can effectively suppress regeneration of other species, including sugar maple, together with beech preferentially filling newly created canopy gaps (Bose et al. 2017a, 2017b; Leduc et al. 2024). Thus, beech proliferation in the understory occurs following the formation of canopy openings and regeneration release (Nolet et al. 2008; St‐Jean et al. 2021), but the response can originate from extensive root suckering by declining mature beech trees in response to infection by beech bark disease (Kish et al. 2022). Unlike beech, sugar maple has no avenue for habitat expansion through root suckering, given that it is not capable of this mode of vegetative reproduction (Godman et al. 1990).

Gradual shifts from sugar maple to beech dominance result in diminished deposition of nutrient‐rich maple leaves and gradually decreased litter decay rates within stands (Jacob et al. 2010; Midgley et al. 2015), stemming from increasing inputs of tougher, lower‐quality leaves (increasing C:N or lignin:N) of beech, accompanied by lower soil pH, decelerated N‐cycling, and N‐availability. Other effects of maple to beech transitions include greater forest‐floor accumulations, higher fungal:bacterial ratios, and a shift from inorganic‐N to an organic‐N economy (Midgley et al. 2015). Increasing beech dominance places at risk valuable ecosystem goods (syrup and high‐value timber) and services (biogeochemical cycling) that are provided by sugar maple, particularly at the northern limits of its range (Boakye et al. 2023).

Annual mass loss rates (*k‐*values, year^−1^) of sugar maple are intermediate among rates of broadleaf or conifer litters encountered within northern hardwood stands (Côté and Fyles 1994; Gartner and Cardon 2004; Midgley et al. 2015). The nutrient‐rich character of maple leaves, together with other litter traits, can vary with latitude, forest type, topographic, edaphic, and hydro‐ecological properties (Tauc et al. 2020), and parent material (Yanai et al. 2012), which further shape patterns of litter decomposition (Bélanger et al. 2019; Mori et al. 2020). These same factors define broader communities of plants and their associated rhizospheres and mycorrhizae and govern not only the disappearance of their dead tissues but also their modes of nutrient acquisition (Midgley et al. 2015). Temperature and moisture drive early phases of litter decay, including those of sugar maple and American beech, together with litter traits such as substrate quality and decomposer communities (Mori et al. 2020; Canessa et al. 2021). What is not known, however, is the relative importance of their contributions throughout the process of decomposer‐driven degradation of readily identifiable tissues and metabolites (i.e., decreasing structural and chemical complexity) and their conversion along the pathway to more amorphous soil organic matter (SOM) fractions through active microbial synthesis (i.e., increasing incorporation and stabilization within the mineral soil matrix). Effects of drought on more fundamental processes in the boreal biome and adjacent northern hardwood forest transition zone, that is, nutrient cycling, largely remain unknown (Houle et al. 2016). However, more widespread evidence has been steadily accumulating by using precipitation shelters (Homyak et al. 2017; Jourdan and Hättenschwiler 2021; Quer et al. 2022; Courcot et al. 2024; Knapp et al. 2024).

Factors controlling litter decay at its debut certainly will differ from those controlling later stages of transformations to humus (Prescott 2010; Moore et al. 2017). Moreover, such drivers and their interactions can be modified or masked by disturbance effects, which range from local to regional scales. Disturbances may be anthropogenic in origin, that is, fossil‐fuel burning, acid precipitation, and N‐deposition, despite substantial North American emissions reductions, urbanization, and forestry operations (Roy, Surget‐Groba, Delagrange, and Rivest 2021; Roy, Surget‐Groba, and Rivest 2021; Prescott and Vesterdal 2021). Conversely, intermittent perturbation stresses may reflect and amplify ongoing climate change and associated environmental processes worldwide, that is, recurrent droughts and wildfires of increasing frequency and intensity, habitat loss, species migration, and range shifts (Boisvert‐Marsh et al. 2019; Rapp et al. 2019; Carteron et al. 2020), and pest/pathogen outbreaks (Kish et al. 2022). Consistent with frequent summer drought in North America, which is recurring more commonly in these high‐latitude forests (Martin‐Benito and Pederson 2015; Moreau et al. 2020), we incorporated experimentally induced soil moisture deficiencies as a principal treatment manipulation into a short‐term factorial litter decay experiment (Phase 1, leaching; Prescott 2010). Rainfall manipulations have a long history of use in assessing vegetation responses and physiological functions to moisture limitations in the field, together with microbially mediated processes such as decomposition (Gavinet et al. 2019; Yu et al. 2019; Quer et al. 2022).

Our experiment was conducted during summer 2021 and 2022 in sugar maple‐dominated forest stands of the Kenauk Reserve (Quebec, Canada). American beech saplings were present within the understory of half of the stands, while absent or sparsely represented in the other half. We present results of a very short‐term study where litter bags of three increasing mesh sizes, each containing only leaves of sugar maple, only leaves of American beech, or a 50:50 leaf mixture of the two species, were incubated in the forest floor of sugar maple stands with and without beech sapling proliferation in the understory (Table 1).

**TABLE 1:** Canopy and understory composition of the six experimental sites, together with their respective basal area (BA, m^2^ ha^−1^) estimates, Kenauk Nature Reserve, QC. The plot inventories were conducted in 2021 and revised in 2024. Total canopy BA% as sugar maple and beech sapling as % of total sapling BA are included in parentheses. Shade‐tolerance ratings (Leak et al. 2014) were weighted across the individual BAs of tree species in the canopy.

Site	Plot	Beech	Exclude	Total canopy BA (%)	Canopy tree species	Shade rating	Total sapling BA	Beech sapl. BA (%)	Sapling species
1	1‐1	Yes	Yes	35.29 (57.89)	ACSA, FAGR, ACRU, BEAL, TSCA	1.44	3.28	3.11 (95.0)	FAGR, ACSA
1	1‐2	Yes	No	35.73 (27.01)	ACSA, FAGR, ACRU, BEAL, ACPE, PRSE	2.55	3.04	2.25 (74.1)	FAGR, ACSA, BEAL
2	2‐1	Yes	Yes	29.18 (93.08)	ACSA, FAGR, ACPE, POGR, OSVI, PRSE, ACPE	1.17	3.23	2.50 (77.4)	FAGR, ACPE, PRSE, OSVI
2	2‐2	Yes	No	40.96 (61.64)	ACSA, FAGR, OSVI, ACPE, BEAL, PRSE. POGR	1.94	2.67	2.38 (89.1)	FAGR, ACPE
3	3‐1	Yes	Yes	25.02 (72.86)	ACSA, FAGR, PRSE, FRAM, ACPE, OSVI	1.65	3.10	3.10 (100.0)	FAGR, BEAL
3	3‐2	Yes	No	31.00 (62.52)	ACSA, FAGR, ACPE, PRSE	1.90	2.49	2.36 (94.8)	FAGR, ACPE
5	5‐1	No	Yes	34.64 (76.13)	ACSA, TIAM, FRAM, OSVI	2.19	0.93	0.0 (0)	OSVI, ACSA, FAGR
5	5‐2	No	No	41.04 (93.45)	ACSA, TIAM, OSVI, ACPE	1.56	0.88	0.0 (0)	OSVI, ACSA, FAGR
6	6‐1	No	Yes	30.25 (99.99)	ACSA, FAGR, ACPE, OSVI	1.00	0.37	0.0 (0)	FRAM, ACSA, FAGR
6	6‐2	No	No	32.50 (99.88)	ACSA, ACPE, OSVI	1.01	0.71	0.0 (0)	ACPE, ACSA, ACPE
7	7‐1	No	Yes	36.23 (92.19)	ACSA, OSVI	1.34	0.82	0.0 (0)	FAGR, OSVI, ACSA
7	7‐2	No	No	31.54 (86.66)	ACSA, OSVI, ACPE, FAGR	1.53	0.06	0.02 (33.3)	FAGR, OSVI, ACSA

*Note:* Plots are 20 m × 20 m; the entire surface is covered under rainfall exclusion. Species from most (1) to least shade‐tolerant (4): ACSA, *Acer saccharum* (1); FAGR, *Fagus grandifolia* (1); TSCA, *Tsuga canadensis* (1); ACPE, *A. pensylvanicum* (1); OSVI, *Ostrya virginiana* (2); ACRU, *A. rubrum* (3); TIAM, *Tilia americana* (3); BEAL, *Betula alleghaniensis* (3); PRSE, *Prunus serotina* (3); FRAM, *Fraxinus americana* (3); POGR, *Populus grandidentata* (4).

Northern hardwood forests contain many more canopy species relative to those found in boreal stands. As such, litter fall and forest floor composition should reflect this variation in overstory composition (Bigelow and Canham 2015) and subsequent litter decay rates. As a canopy dominant, we expected that American beech would affect decomposition processes in a manner similar to species‐diverse European stands that are affected by the presence of *Fagus sylvatica* L. leaf litter (Jacob et al. 2010). We examined shifts in plant community composition, both in the canopy and understory, which were associated with beech sapling proliferation, on leaf litter decay. Indeed, litter decay may be mediated by tree species diversity through litter fall deposition and its spatial mixing and redistribution, together with altering their associated decomposer microbe communities (Laganière et al. 2010; Bigelow and Canham 2015; Beugnon et al. 2023). We posited that maple litter would decay more slowly within a forest‐floor matrix of low chemical quality that had developed with increasing beech litter inputs and where beech saplings proliferated, and vice versa, that is, beech litter would decay more rapidly in maple‐dominated higher quality forest floor accumulations.

Beneath the forest canopy, we assumed that responses of soil moisture would be much more variable than those of temperature among the experimental treatments. These effects were examined through ANOVA, correlation, and regression analyses. In assuming that soil moisture was the predominant environmental driver of decomposition, we also employed physiological time to test the temperature effect, substituting growing degree‐days (GDD) for regular time duration in our regressions.

We focused on three objectives. Objective 1 tested the effects of experimental drought (rainfall exclusion) manipulations that incurred soil‐moisture deficits. In the absence of rainfall entering the soil, we posited that mass loss rates of all three litters would be severely retarded by soil moisture deficits that were incurred over summer‐long incubations, consistent with expectation. Conversely, we expected that increasing summer moisture availability would not slow litter decay in the three leaf litter types that had been deployed; rather, high‐quality maple litter would decay rapidly, while low‐quality beech litter would decay slowly, with the 50:50 maple‐beech mixture losing mass in a manner more reflective of the properties of pure maple with its higher N and soluble matter contents, and lower secondary metabolites (e.g., lignin, phenolics). Concentrations of the nutrients would be dictated, in turn, by the leaf litter pack's mixture ratios (Setiawan et al. 2016; Grossman et al. 2020; see Jacob et al. 2010), while possibly also biasing tests of their additivity.

Indeed, Objective 2 examined the effects of litter mixing on rates of litter disappearance relative to the monospefic litters. According to Ostrofsky (2007), “additivity” assumes that mass‐loss rates averaged over two or more individual species equate to the rate for a mixed assemblage that includes the same species. If this process assumes no interaction, especially when represented as a single‐exponential decay model, inhibition or facilitation of decay by one species over another would otherwise go undetected or underestimated, depending upon the duration of the litter incubation experiment and the magnitude of differences in substrate quality between the species.

Setiawan et al. (2016) have noted that litter mixtures frequently exhibit higher (synergism) rather than lower decay rates (antagonism) compared to their monospecific litters, particularly in the presence of soil fauna. Using conventional test methods, thereby producing a ratio of standardized mean differences divided by pooled variances, we predicted that the two‐species mixture would decay at a rate that was intermediate between the two pure litters (additive effect; Gartner and Cardon 2004). For more comprehensive estimates, we assessed non‐additivity by calculating *k*‐values (mass loss rates) of the individual species in each mixed litter bag, which might indicate bio‐ or physicochemical interactions between dissimilar litter tissues in intimate contact with one another (Ostrofsky 2007).

Objective 3 involved assessments of mesh‐size differences on mass loss (Peng et al. 2022), although the activity of faunal decomposers was not a focus of the study. On the one hand, increasing mesh aperture widths from small (S) to medium (M) to large (L) would deny or allow access to litter by a broad faunal size range that is characteristic of deciduous forests. On the other hand, increasing exposure of confined litter to the broader forest floor environment could increase spillage losses (possibly favoring L > M > S), while possibly favoring altered microclimates within leaf packs (possibly favoring S > M > L). Soil moisture deficits were posited to limit litter decay strongly under rainfall exclusion, regardless of beech proliferation, differences in bag aperture sizes, and substrate quality differences among the three litter types (“Species”). Under rainfall exclusion, all decay rates were assumed to be low and similar among treatments. We assessed the rankings of mass‐loss rates across the aperture sizes and the consistency of their ordering over each of the other treatment combinations (Concordance analysis).

## 2 Materials and Methods

### 2.1 Study Region

The study was conducted in southwestern Quebec (Canada). Experimental plots were established in temperate forest stands within the Kenauk Nature Reserve (45.75°, −74.94°). The 26,500 ha of forests, lakes, and wetlands constituting this privately owned reserve comprise a network of locations that are dedicated to conservation and preservation, research, educational, and recreational activities, together with logging operations (Roy, Surget‐Groba, Delagrange, and Rivest 2021; Roy, Surget‐Groba, and Rivest 2021).

Throughout the northern hardwoods of Quebec, and across temperate North America, forest management is primarily uneven‐aged silviculture favoring stand regrowth with three or more age classes (Nolet et al. 2018). Hardwood forests of the Kenauk Nature Reserve are dominated by deciduous species that are managed according to different regimes, employing both even‐aged and uneven‐aged silvicultural prescriptions, including experimental manipulations (Roy, Surget‐Groba, Delagrange, and Rivest 2021; Roy, Surget‐Groba, and Rivest 2021). Despite the presence of forestry operations within the reserve, these late‐successional stands (+200 years old) have experienced few natural disturbances, that is, outbreaks of insect defoliators ( *Malacosoma disstria* ), thereby remaining largely uneven‐aged. Soils supporting these forest stands originate from glacial till (Roy, Surget‐Groba, Delagrange, and Rivest 2021; Roy, Surget‐Groba, and Rivest 2021), and are classed as Dystric Brunisols in the Canadian system (USDA: Typic Dystrochrepts) with moder‐type humus. Regional climate is cold‐temperate continental. For 1981–2010, average annual air temperature was 5.3°C and annual precipitation averages 1204 mm (station 7035110, Notre‐Dame‐de‐la‐Paix, Environment & Climate Change Canada).

### 2.2 Experimental Design

**FIGURE 1:**
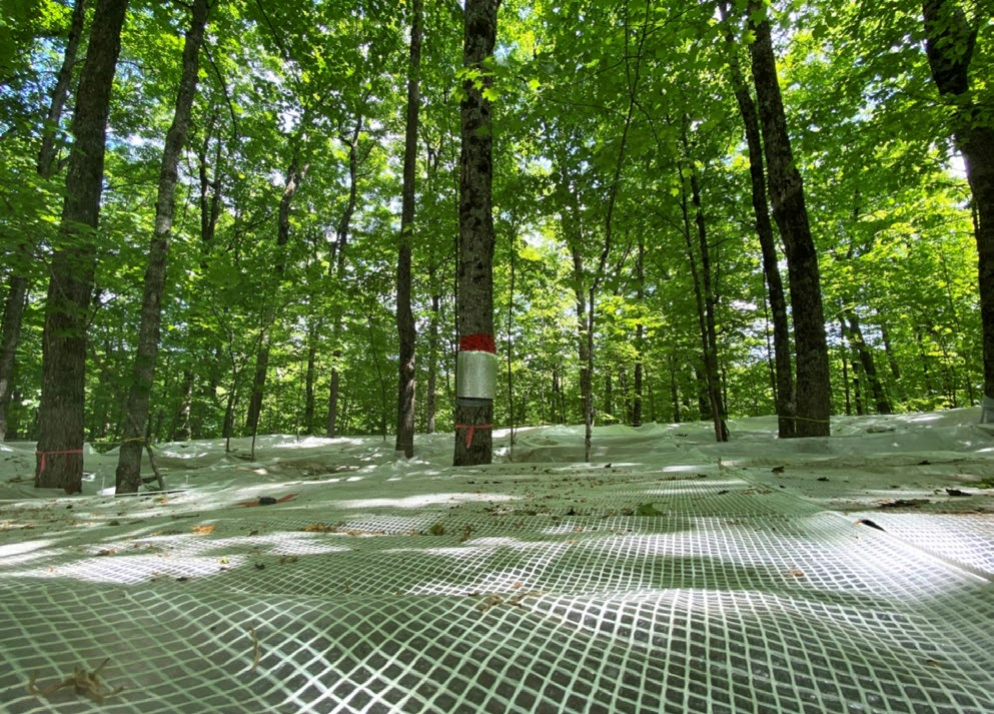
Rainfall exclusion tent as seen from above a 20 m × 20 m plot, one of 6 covered and 6 open plots that are located in maple‐dominated northern hardwood forest stands, Kenauk Nature Preserve (Quebec, Canada), summer 2023. Overlapping impermeable tarps forming the tent are suspended from trees at 1.3 m above the ground surface across the entire plot (Photo credit: Audrey Maheu).

Six neighboring stands were selected for the rainfall exclusion experiment, in which sugar maple dominated the canopy that was not subject to recent silvicultural prescriptions (Table 1). American beech proliferated in the understory in three stands (Sites 1, 2 and 3), while three stands were essentially beech‐sapling‐free (Sites 5, 6, and 7). Each site contained two 20 m × 20 m plots to manipulate soil moisture, that is, with or without precipitation interception and diversion. Definitive changes in the hydrologic context of decomposition, that is, cumulative soil‐water deficits, were thus imposed on six of the 12 plots by deploying rainfall excluders. Excluder tents were erected for the entire summer. They were deployed for two consecutive years: first, from early July to mid‐September 2021 and second, from June to mid‐September 2022. The tents were disassembled prior to the intervening winter period (2021–2022). Summer rainfall was intercepted by the excluders (> 75%) and drained from the plots. The designated 20 m × 20 m exclusion plots were completely covered with clear polyethylene tarps (Kleton, 8 mil‐thick, 3.6 m × 3 m; Tenaquip Ltd., Senneville, QC). Tarps were deployed to form the tent that was suspended from the trees within the plot at ~1.5 m above the ground surface (Figure 1).

Having set the experimental design, we refer throughout the paper to the first natural treatment factor of beech sapling proliferation as “Proliferation,” where the two levels (plots) in which “Beech Proliferation” has occurred are coded as “Yes,” versus plots where dense beech sapling proliferation has not occurred, which are coded as “No” or often denoted as “beech‐free.” The second treatment factor, an experimental manipulation of rainfall inputs, is denoted “Rainfall exclusion” and has two levels. These levels are coded as “No rainfall” (Exclusion = Yes) versus “Rainfall‐accessible” or “Rainfall” (Exclusion = No). Crossing two levels of proliferation with two levels of exclusion resulted in four treatment combinations of the fixed factors.

Each treatment combination was replicated across three sites; site was a random factor and resulted in 12 plots (Table 1). During the litter bag experiment (June–September 2022; see following section) and in the previous year (June–September 2021), summer readings of mineral soil temperature and volumetric moisture content (TDR readings, see below) were logged continuously (15 min intervals) in six plots where rainfall reached the forest floor (Exclusion: No) and six in which rainfall was intercepted and diverted before reaching the ground (Exclusion: Yes). Lengthy data records were accumulated over two consecutive growing seasons; we describe only general trends (see Section 2.4).

### 2.3 Litter Incubation Study

Litter incubations were performed in the 12 plots between 20 June and 17 September 2022 using fiberglass mesh bags. Short experimental duration coincided with rainfall excluder placement. Senesced sugar maple and beech leaves were collected in late October 2021 within six beech‐dominated plots. Leaf collections were mixed, then sorted by species and oven‐dried (48 h at 60°C). Experimental treatments were expanded beyond proliferation and exclusion to include three litter types (here, denoted “Species”) and three litter bag mesh sizes. Litter was deployed in 20 × 20 cm bags, that is, monospecific maple (3 g) and monospecific beech (3 g), and a 50:50 mixture of the two species. Initial dry mass (3 g aliquot) of pure maple or beech litter was weighed into each litter bag with an analytical balance (±0.001 g). In mixed litter bags, initial leaf masses consisted of 1.5 g of sugar maple and 1.5 g of beech.

The three litter types were crossed with litter bags that were constructed from mesh cloth with three aperture sizes (Peng et al. 2022). Fine mesh includes microbes and soil micro‐fauna (< 0.1 mm diameter), yet physically excludes meso‐ and macro‐fauna (0.1–20 mm dia.); medium mesh apertures exclude macro‐fauna (> 2 mm dia.); and a large mesh excludes mega‐fauna (> 20 mm dia.), that is, burrowing vertebrates or large invertebrates. The treatment is denoted Meshcode, but its levels may be referred to as Small, Medium, and Large. The mesh‐size range permitted entry of smaller detritivores that contributed to litter decay (grazing, fecal inputs), while also indicating whether fine mesh bags altered leaf pack microclimate by increasing moisture retention or temperature and hindering faunal colonization (Bokhorst and Wardle 2013). Large meshes encourage leaching and light penetration, while possibly overestimating decay rates through increased spillage. Despite the inclusion of different meshes, actual assessment of faunal communities in the plots was beyond the scope of the study. Furthermore, we did not record temperature or moisture conditions within the bags.

During litter bag deployment (June 20, 2022), care was taken to insert them beneath the forest floor (depth of 1–2 cm), in intimate horizontal contact with the underlying mineral soil. Moder humus is generally thin (F‐H layer = 2–4 cm), but this attribute and the L‐layer were not formally measured in each plot, nor was other micro‐site variation assessed. Bags of three mesh sizes (Meshcode) were installed as a set of grouped triplets for each of the three “Species” litters (maple, beech, mixed) at random locations in each plot. The original design called for deployment of duplicate bags (“Replicates”) of these nine combinations in the paired Exclusion plots (Rainfall vs. No Rainfall), which increased the number of experimental observations and the statistical power of our models. Sufficient litter bag deployments required that 3 Species × 3 Meshcode × 2 Exclusion × 2 Replicates × 2 Proliferation × 3 Sites × 3 Months, or 648 bags be deployed to complete the experimental treatments. “Replicates” was subsumed into the model error term.

Bags were removed on three dates, that is, July 19, August 18, and September 17, 2022. Removal avoided spillage losses during litter bag transport to the laboratory in re‐sealable polyethylene bags stored within an ice‐chest. Samples were cleaned of extraneous debris and sorted to species (mixed bags) for each monthly retrieval, that is, 30, 60, and 90 days. Very little mineral soil contaminated the litter (C. Ghotsa Mekontchou, personal observation). Cleaned litter was dried for a week under circulating airflow. Air‐dried material was then oven‐dried at 60°C for 72 h, after which its mass was recorded on an analytical balance (±0.001 g). These data were used to determine mass loss rates, once they were converted to percentages of initial mass (3 g) and Ln‐transformed for regression. We also calculated instantaneous decay estimates for every bag as ([Ln‐remaining% − Ln‐100%]/year‐fraction) from each litter sample; separate *k*‐estimates were also made for each species within the mixed bags, based on initial 1.5 g masses. (Given unforeseen circumstances, processing of litter and calculation of its mass losses was not completed for Site 3.)

### 2.4 Characterizing the Physical Environment

We conducted inventories of the six forest stands within the two 20 m × 20 m plots established on each site. Individual trees > 9 cm DBH (diameter at breast height, 1.3 m) were counted in each plot to estimate density (stems ha^−1^). Respective plot basal areas (m^2^) were recorded for each species that was identified (Table 1) and standardized to a per‐hectare basis. Plot understories (saplings, DBH < 9 cm) were recorded by species in 100 m^2^ quadrats. Species data were combined to estimate total overstory (tree) and total understory (sapling) basal area (m^2^ ha^−1^). Inventories were conducted in 2021 and revised in 2024 to gauge vegetation differences across the experiment.

We measured several soil properties across plots. Mineral soil (0–15 cm) was composited by plot and air‐dried before sieving to pass a 2.0‐mm mesh. Following screening, 40–50 g aliquots were treated with 30% H_2_O_2_ to remove SOM, dried, and dispersed in aqueous hexa‐metaphosphate. For textural analyses, sand, silt, and clay fractions in the suspensions were determined by hydrometry. Fermentation‐Humification (F‐H) layer samples were transported back to the laboratory and dried for pH. Mineral soil pH was measured electrometrically on slurries of the mineral fraction (10 g soil:20 mL deionized water), while F‐H pH was measured on 1:7 slurries (10 g organic material:70 mL deionized water), likewise using a pH meter equipped with a combination electrode. To assess rainfall exclusion effects while capturing spatial variability (see below), multiple measurements of soil water content (SWC) were taken in the plots near the end of the second field season (1 September 2022). We made these measurements in lieu of micro‐site assessments that would have characterized pit‐and‐mound micro‐topography and root system distributions (Bélanger et al. 2019). In situ summer readings were logged continuously (15 min intervals) in six plots that intercepted rainfall (Exclusion: No) and six where rainfall did not reach the ground (Exclusion: Yes) for the summers of 2021 and 2022. Given lengthy records that were accumulated (5000–8000 readings per logger) over each of the two consecutive growing seasons, we describe overall trends.

Portable TDR (Time‐Domain‐Reflectometry) that was equipped with 12‐cm‐long wave‐guides (FieldScout TDR 100, Spectrum Technologies Inc., Aurora, IL) was employed in manual measurements. Wave‐guides were fully inserted vertically into the mineral soil (12 cm depth) for integrated SWC measurements. In excluder plots, we took three averaged measurements at each of 42 positions within a 6 row by 7 column grid, under rainfall‐impermeable tarps, which were arrayed to form a tent across each exclusion plot. The 6 × 7 grid required roughly three‐meter spacing between grid nodes. We reproduced this same grid pattern in plots without rainfall excluders, taking SWC for 6 rows by 7 columns. An exemplar of this grid layout is presented for Site 1‐1 (Rainfall excluder present) in Appendix 4. For each plot grid, means and standard deviations (±SD) were estimated from 42 readings; percentage coefficients of variation (CV%) for each plot were calculated from these estimates. We also estimated CVs for the three measurements at the individual grid nodes, but they were not subjected to further analysis.

### 2.5 Statistical Analysis

The litter bag incubation dataset was incomplete, given the limited entry of the Site 3 excluder plot results (see Section 2.3). Therefore, we excluded all Site 3 mass loss data from the decomposition analysis. This left the random effect unbalanced (Sites 1 and 2, vs. 5, 6, and 7). Preliminary analyses were conducted on instantaneous *k*‐values that were obtained from the 30, 60, and 90 day incubation periods (Time) to determine which factors most strongly influenced litter decay responses. We constructed a linear mixed‐effects model incorporating rainfall exclusion (2 levels), beech proliferation (2 levels), meshcode (3 levels), and species (3 levels) as fixed effects, with the remaining five sites as random effects.

Mixed‐effects model terms were estimated by Restricted Maximum Likelihood (REML) and tested with the Satterthwaite method in JASP (JASP Team 2020, Version 0.13; [Computer software]). Differences among treatment means for individual factors were assessed using Holm post hoc tests. Homoscedasticity could not be confirmed for raw data (Levene's test: *p* = 0.013). However, visual inspection of *Q*‐*Q* plots suggested normal model residuals. An ANOVA summary of linear mixed‐effects modeling is included in Appendix 1.

Other relevant ANOVAs or correlation analyses using instantaneous *k*‐values or final mass‐remaining estimates were restricted to 90 days, given that the very short‐term field estimates that were obtained for 30 or 60 days are likely unreliable with respect to the former (Prescott and Vesterdal 2021). Indeed, estimated single‐pool exponential *k*‐values cannot be assumed to be constant over time, even though this may often be a naive simplifying assumption, particularly in the multiple regression model that we used (see below), whereas multi‐compartmental models or non‐linear formulas like the Weibull distribution may be more realistic approximations of litter behavior, but offer less tractable solutions (Cornwell and Weedon 2014). Moreover, estimates may be inconsistent, given that they were possibly subject to strong month‐to‐month variation in soil moisture and temperature (Yu et al. 2019).

We presented only 90 day results, but ranked the mass loss responses across 12 treatment combinations separately for each monthly removal period. The treatments are coded as follows: Yes‐Yes‐Maple; Yes‐Yes‐Beech; Yes‐Yes‐Mixed; Yes‐No‐Maple; Yes‐No‐Beech; Yes‐No‐Mixed; No‐Yes‐Maple; No‐Yes‐Beech; No‐Yes‐Mixed; No‐No‐Maple; No‐No‐Beech; and No‐No‐Mixed. We expected progressive loss in mass over the course of the litter incubation. We ranked mean mass remaining over the consecutive removal dates (i.e., 30, 60, and 90 days) for each of the 12 treatment combinations (blocks), determining their consistency using Kendall's Coefficient of Concordance (*W* = 1, complete agreement among blocks; *W* = 0, no agreement among blocks). Likewise, consistency of mass loss rates across large, medium, and small mesh sizes was determined at 90 days for the same 12 treatments using Kendall's *W*. We also compared the ordering of mass losses at 90 days as a Spearman correlation (*r*_s_) with *k*‐values that were estimated by multiple regression using a general linear model (GLM), which is described below.

Under GLM, ln‐transformed percentage mass‐remaining was regressed against incubation period, the latter expressed in years rather than in days (*y* = 0.0822, 0.1644, and 0.2466). The linear form of Olson's (1963) negative exponential model was extended to multiple treatment groups: ln*M*_t_ = ln*M*_0_ − *kt*, where *M*_t_ is net mass‐remaining (%) at time *t* (years), *M*_0_ is initial mass (100%), and *k* is the regression slope coefficient (year^−1^). We extended this model to estimate mass‐loss rates (*β*) for 12 treatment combinations (Appendix 2). The model was also run using growing degree‐days (GDD, base 5°C) as the predictor to test the effect of temperature as a driver of decay. Support for candidate models during model selection was provided by Bayesian analysis in JASP, assuming naïve priors for the predictors (*p* = 0.5).

The multiple regression model not only allowed us to use the full mass‐loss dataset, but also to include plot‐level continuous variables for each incubation period. These included: (1) growing degree‐days (GDD, 5°C threshold) that were extracted from the continuously logged soil temperature data, where GDD replaced year‐fraction in the model; (2) basal area of *Acer* species in the canopy other than very shade‐tolerant *A. saccharum* ; (3) basal area of saplings; (4) monthly gain/loss of soil moisture (cm^3^ cm^−3^ 30 day) that was estimated from the difference in SWC between the beginning and end of each 30‐day incubation period (gain/loss per day) and multiplied by 30 days; (5) spatial variability in final SWC using plot CV%; and (6) mean soil pH. (7) We further considered overstory species richness as a possible factor influencing the composition of the forest floor and subsequent litter decay. For each plot, we averaged shade‐tolerance ratings of the canopy species (see Table 1) each weighted by their respective canopy species basal area. A more direct assessment of forest floor composition and litter production would have been desirable, but these data were not available.

**FIGURE 2:**
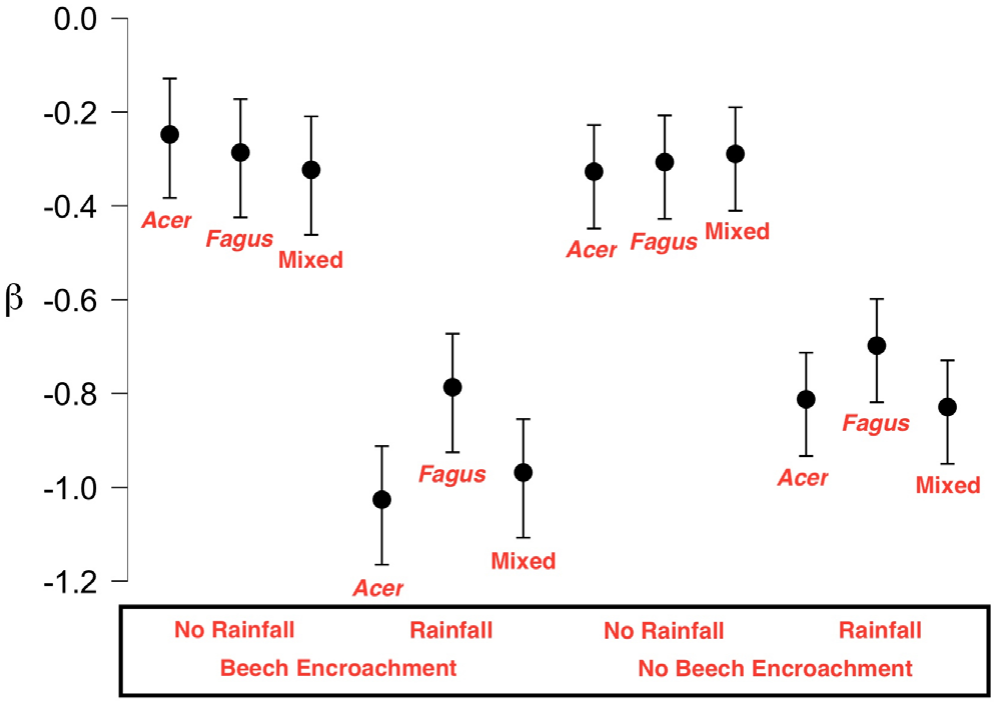
Estimated decomposition rates (*k*‐value, year^−1^) of monospecific “Maple,” monospecific “Beech” and 50:50 mixed leaf litter (“Mixed”) that were averaged over mesh sizes and which were incubated for up to 90 days under moisture limitation (Exclusion: No Rainfall) and under no moisture limitation (Exclusion: Rainfall accessible), in the presence (Proliferation: Yes) or absence (Proliferation: No) of beech saplings. Error bars associated with the decay rates (*β* slope coefficients) are 95% credible intervals. Estimates are based upon Bayesian multiple regression results that are summarized in Appendix 2.

Differences between averaged monospecific litter decay rates (= [maple‐*k* + beech‐*k*]/2) and observed mixed litter decay rates that were derived from the GLM were tested for additivity, and subsequently inspected for signs of non‐additivity using their 95% credible limits, depicted in Figure 2 and Appendix 2. Standardized differences (= [observed − expected]/expected) were calculated between the mixed litter and averaged individual species rates (expected values, Appendix 2); we divided the squared mean differences by their pooled variances to obtain *Z*‐scores. We followed Ostrofsky's (2007) advice that separate estimates should be made for mass loss rates of maple and beech litter within the 50:50 mixed litter bags, given that combining two extreme litters (high labile content vs. low labile content) may unduly bias *k*‐value estimates at different times during the decay process. Mean estimates were obtained from ANOVA on 90‐day instantaneous‐*k* in response to Proliferation, Exclusion, Species, and Meshcode effects. We determined whether these estimates fell within the 95% credible intervals for slope coefficients (*k*‐values) of the monospecific bags determined in the multiple regression (Appendix 2).

Differences in vegetation and soil properties between Exclusion and Proliferation treatments were tested using PERMANOVA. One‐ or two‐way PERMANOVA (9999 permutations) was executed in PAST version 4 (http://palaeo‐electronica.org/2001_1/past/issue1_01.htm). It was applied to both plot‐level univariate (total canopy tree basal area and total sapling basal area) and multivariate (individual tree species basal area) data prior to the implementation of the rainfall exclusion treatment. We tested the univariate hypothesis: total BA would not differ between stand canopies under which beech saplings proliferated or which were beech‐free (Yes vs. No). Total sapling area was assumed to differ between the two understory proliferation conditions (Yes vs. No) for obvious reasons. For the multivariate test of community differences (PERMANOVA), we hypothesized that canopy tree basal areas of the nine most common species would not differ among the plots in sites where beech saplings had proliferated (Yes) versus beech‐sapling free (No), as summarized in Table 1. Exclusion was not entered into the model, given that the inventories were conducted prior to the implementation of the experimental manipulation. Two rare species were excluded (FRAM, TSCA). Shade‐tolerance ratings devised by Leak et al. (2014) were used to rate the canopy of each plot by weighting each tolerance rating by tree BA contributed by each species. A Mann–Whitney *U*‐test compared shade‐tolerance indices of beech‐free versus beech‐proliferated sites.

One‐way PERMANOVA similarly tested soil textural differences between plots on which beech proliferated and where plots were beech‐free. Soil textural fractions (three dependent variables: Sand %, Silt %, Clay %) were brought into Euclidean space by center‐log‐ratio (*clr*)‐transformation (PAST 4). Manual readings of SWC (0–12 cm) that were taken at the end of the study were analyzed over the 6 × 7 cell grid on each plot. We focused on 12 mean SWC plot estimates and their coefficients of variation (CV%), each followed by two‐way PERMANOVA. We expected mean SWC to be low under excluders (see Table 3), yet we also expected that soil moisture would spatially vary strongly and unpredictably as indicated by their associated CV%. In plots with access to precipitation, SWC means would be higher than in plot soils under excluders, while also exhibiting greater uniformity of moisture distribution across their surfaces (i.e., lower CVs). As previously stated, we used these soil moisture variability (CV%) data as an additional covariate in the litter decay GLM.

## 3 Results

### 3.1 Rainfall Exclusion Dominated Effects on Instantaneous Litter Mass Loss Rates

Mixed‐model analysis of instantaneous‐*k* (Appendix 1) shows that rainfall exclusion exerted the strongest effects upon litter decay, followed by mesh‐size, species, and exclusion × species. Beech proliferation affected mass loss rates, but only through weak interaction with exclusion. Post hoc tests indicate that exclusion (Reference condition: No rainfall) reduced litter decay rates two‐ to three‐fold (mean *k*: −0.321 year^−1^) compared to excluder‐free conditions (−0.870 year^−1^). Against large mesh as the reference, medium‐mesh decreased rates slightly (−0.542 year^−1^), but decay in fine‐mesh bags (−0.573 year^−1^) was not appreciably affected. For species, beech litter decayed more slowly than the reference (maple: −0.532 year^−1^), while mixed litter decay was similar to pure maple (−0.608 year^−1^). With respect to interaction, only beech litter decayed more slowly than maple or mixed litter (−0.534 year^−1^) under excluder‐free conditions.

Rankings of 90‐day mass‐remaining estimates over the 12 treatment combinations (Table 4) were highly consistent with rankings for the 30‐ and 60‐day responses (Very good agreement: Kendall's *W* = 0.927, Chi‐square = 22.24, df = 11, *p* = 0.012). Furthermore, mesh‐size effects over the 12 treatments indicated no consistent agreement among ratings for large, medium, and small mesh apertures (Poor agreement: *W* = 0.116). The greatest mass‐loss rates were observed for large‐mesh bags in five treatments, while medium and small meshes accounted for four and three treatments, respectively (Table 4). Mixed litter in beech‐free plots that were covered with rainfall excluders decayed at similarly low rates across mesh sizes.

Plot‐level factors that are correlated with instantaneous *k*‐estimates included soil moisture (Table 3, Sites 1, 2, 5, 6, and 7; Spearman correlation: *r*_s_ = −0.952, *p* < 0.001), consistent with initial expectations. Furthermore, variation in *k*‐values was positively correlated (*r*_s_ = 0.776, *p* = 0.005) with within‐plot variation in SWC (CV%). The pH in mineral soil and F‐H layer was not correlated with *k*. The low percentage mass loss (6.5%–8.5%) in the 90‐day removal suggested that litter bags under moisture‐limited decay remained in the leaching phase (Table 4). Mass loss that was not constrained by moisture limitation averaged from 15.5% to 24.4% (Table 4). At 90 days, leaching losses from maple and beech mass in the mixed bags, when they were compared with those of monospecific bags, did not differ (*p* > 0.05), but this result may be due to small sample sizes that were involved. These individual losses were small as well, also likely indicating Phase 1 decay, that is, the leaching stage.

### 3.2 Mass Loss Rates Estimated by Multiple Regression Differ With Rainfall Exclusion or Inclusion

Slope coefficients that were derived from multiple regression for the 12 Species‐Proliferation‐Exclusion combinations each differed from zero (Appendix 2, 95% credible intervals). We did not report Bayesian Factors (BF) indicating support for the model, but they greatly exceeded 1 (range: 2181.5, 2.651e + 41), thereby very strongly supporting the hypothesis *β*_*i*_ ≠ 0. Except for the *Y*‐intercept (= 4.506 or 90.1%), regression results have been depicted in Figure 2. Collectively, mass loss rates of litter bags (Appendix 2) that were incubated under moisture limitation (No Rainfall) were 2.88 times slower (mean *k* = −0.296, CV = 9.8%) than for bags that were not subject to moisture limitation (mean *k* = −0.854, CV = 14.2%). Examination of both decay rates (Figure 2, Appendix 2) and final masses (day 90) of maple, beech, and mixed litters (Table 4) suggested that 50:50 mixtures behave more like maple alone compared to beech alone, but only under freely accessible precipitation.

Our original regression model explained 54.8% of variation in Ln‐mass remaining (adjusted *R* ^2^ = 54%). When we reran the regression substituting GDD_5 for year‐fraction as the predictor, the model only explained about 53.5% of variation in the data (not shown). Furthermore, a plot (not shown) including estimates of the 12 slope coefficients and their credible intervals closely resembled the patterns that we obtained in Figure 2; therefore, we did not explore the relationship between decay and temperature any further. The initial time‐based regression was also rerun to include additional individual plot‐level predictors, potentially improving decay rate estimates. Five remaining predictors were used individually, but each only increased the explained variance by 1%–2%. Furthermore, 95% credible intervals around their coefficients included zero, except for BA for *Acer* species other than ACSA (*β*_13_ = −0.003, [−0.005, −0.0008]). Inclusion of all plot‐level predictors in the regression resulted in a modest increase in variance explained (*R* ^2^ increased to 56.5%). Only slope coefficients for three of these variables were “significant” and retained. These included weighted shade tolerance (*β*_13_ = −0.012, 95% credible interval [−0.023, −0.00004]); volumetric water content change (*β*_14_ = −0.021, [−0.040, −0.005]); and total sapling BA (*β*_15_ = 0.013, [0.002, 0.023]).

### 3.3 Litter Mixture Effects Are Additive, Perhaps

Similar litter decay rates were estimated between beech sapling‐free maple stands and those stands where beech saplings proliferated. In our original GLM, maple litter alone decayed about 26% (standardized difference = [−1.026 + 0.812]/−0.812) faster in the plots containing beech saplings compared to the beech‐free plots; beech litter decayed about 13% faster (= [−0.787 + 0.698]/−0.698) under profuse beech‐sapling cover than under beech‐free conditions. The 50:50 litter mixture likewise decayed more rapidly in the presence of beech saplings, that is, 17% higher (= [−0.969 + 0.829]/−0.829) than in its absence (Appendix 2).

When decomposition is not constrained by moisture limitation (Rainfall exclusion = No), beech litter alone decayed more slowly than did either maple alone or mixed maple‐beech litter. In the original model, the behavior of rainfall‐accessible mixtures conformed to assumptions of additivity. Under beech sapling proliferation, the difference in the 50:50 mixture between observed and expected decay rates (respectively, −0.969 and −0.907 year^−1^) did not differ from zero (*Z* = 0.92). In the absence of beech saplings, the difference in the 50:50 litter bag mixture between observed and expected decay rates (respectively, −0.829 and −0.755 year^−1^) likewise did not differ from zero (*Z* = 1.75). Yet, as decay proceeds, non‐additive effects may emerge in this latter case, given that the mean mass loss rate of the mixture (−0.829 year^−1^) exceeded that of maple alone (−0.812 year^−1^).

Instantaneous *k*‐values were estimated for the 90‐day percent mass‐remaining of each species in the mixed bags, revealing slightly different responses. When mixed bags were incubated in rainfall‐accessible plots, maple decayed 27% (= −1.031/−0.812) and beech decayed 21% (= −0.950/−0.787) more rapidly than did the same litter that had been incubated in monospecific bags. When instantaneous *k*‐values were estimated for each species in mixed bags that were incubated in rainfall excluder plots, maple decayed 58% (= −0.393/−0.248) more rapidly than did the same litter in monospecific bags. Further comparisons of these rates suggested that mixing had a salutary effect on maple mass loss rates when it was incubated in plots where beech proliferated, but less so in the beech‐free plots (Appendix 2). Indeed, synergistic responses would indicate non‐additivity, as would antagonistic responses, including beech that was incubated in mixed bags under excluders, in the absence of beech saplings (Appendix 2). Mixed beech litter decayed 32% more slowly than beech incubated alone (= −0.207/−0.307). Three instantaneous *k*‐values (in boldface) lie beyond 95% credible intervals that are established for multiple regression coefficients of the pure litters. Yet, the negative estimate could not be declared “significant,” given that it still fell within credible intervals for the original multiple regression coefficient. We also found that correlations of 90‐day mass‐loss rates versus weighted shade‐tolerance indices were strong across sites where beech saplings had proliferated (Yes: *r* = −0.740, *p* < 0.001) compared to their absence (No: *r* = 0.008, *p* = 0.934). What the former response indicated was that decay rates were lowest under canopy species with the greatest shade tolerance (i.e., 1) and increased as shade intolerance rankings increased toward 5, consistent with our initial prediction. Instantaneous‐*k* was weakly correlated with weighted shade‐tolerance indices in rainfall‐accessible plots (Exclusion, No: *r* = −0.332, *p* = 0.002), but was uncorrelated under rainfall exclusion (Yes: *r* = −0.091, *p* = 0.392).

### 3.4 Understory Maple BA Varies With Beech Sapling Proliferation, but Not Total Overstory BA

Comparisons of total canopy tree basal area indicated no difference between the two levels of beech sapling proliferation (*p* = 0.924), nor did canopy beech BA vary between these treatments (*p* = 0.291). Mean canopy tree basal area (±SD) is 33.62 (±4.67) m^2^ ha^−1^ across plots (Table 1). Total sapling BA was five‐fold higher in plots (Pseudo‐*F*_1,10_ = 27.27, *p* = 0.0017) where beech saplings have proliferated (2.65 ± 0.36 m^2^ ha^−1^) compared to beech‐free ones (0.51 ± 0.29 m^2^ ha^−1^). Understories in three sites with high sapling densities consisted mainly of beech (Proliferation, Yes: 74%–100%; Table 1), and contained practically no sugar maple. Across six sites, sapling basal areas of sugar maple and beech were strongly and inversely correlated (*r*_s_ = −0.888, *p* < 0.0001). Even in the absence of beech saplings, basal area of sugar maple saplings was low (range: 0.02, 0.88 m^2^ ha^−1^).

Nine tree species (11, including rare spp.) formed the overstories of the 12 plots (Appendix 3). As shown by their mean (±SD) BAs, sugar maple (ACSA) dominated the canopy, followed by beech (FAGR), which was also very shade‐tolerant (rating = 1). The sole conifer that occurred infrequently in the canopy, *Tsuga canadensis* (L.) L. Carrière (TSCA) is very shade‐tolerant, as is striped maple, *Acer pensylvanicum* L. Yet, the latter species (ACPE) occupied less of the canopy according to its basal area than did red maple, *Acer rubrum* L. (ACRU), which is intermediate in its shade tolerance (rating = 3).

The remaining species in our table ranged from shade tolerant to intermediate to very shade intolerant. In terms of BA contributions of the nine species to differences in canopy composition, plots were clearly separated according to beech sapling presence versus absence (Pseudo‐*F*_1,10_ = 9.308, *p* = 0.0022). Effects of Exclusion and Exclusion × Proliferation that were produced in a two‐way version of the model (not shown) did not contribute to differences in canopy composition (PERMANOVA: *p* ≥ 0.393). Our basal area‐weighted estimates of canopy tree shade tolerance in each plot did not differ between levels of beech sapling proliferation (Table 1; *p* = 0.230). Basal area contributions of canopy species were summarized in Appendix 3, together with their mycorrhizal status and shade tolerance rating. Basal area of the individual canopy species differed between beech‐free plots and those where beech saplings proliferated (PERMANOVA, univariate responses: ACSA, BA lower under exclusion; FAGR, BA higher under exclusion; and OSVI, BA lower under exclusion; Appendix 3).

### 3.5 Sandy Loam Texture Does Not Affect Stand Composition, but Plot pH Is Lower With Beech Saplings

Mineral soil beneath the trees is a sandy loam across the six sites, given that there were no differences between the two levels of sapling proliferation (*p* = 0.763). Its overall mean (±SD) textural fractions are summarized in Table 2. Bulk pH of the F‐H layer and mineral soil (Table 2) was strongly correlated across sites (*r* = 0.844, *p* < 0.001). Beech sapling proliferation lowered pH of the two horizons by about one unit relative to beech‐free controls (Pseudo‐*F*_1,10_ = 8.584, *p* = 0.02). Mean (± SD) pH values in the F‐H layer were 4.09 ± 0.35 (prolific beech saplings) versus 5.03 ± 0.48 (beech sapling‐free), respectively. In mineral soil, the respective pH values were 4.16 (±0.20) versus 4.91 (±0.38) for plots with beech sapling proliferation versus beech‐free plots (Pseudo‐*F*_1,10_ = 18.14, *p* = 0.0067).

**TABLE 2:** Bulk pH (in water) of F‐H layer (1:7 m/v slurry) and mineral soil (0–15 cm; 1:2 m/v slurry), together with percentage sand, silt, and clay (determined by hydrometry).

Site	Plot	Beech	Exclude	F‐H pH	Min. pH	Sand (%)	Silt (%)	Clay (%)
1	1‐1	Yes	Yes	4.45	3.90	54.9	42.4	2.6
1	1‐2	Yes	No	3.93	3.99	54.7	41.6	3.6
2	2‐1	Yes	Yes	4.47	4.35	57.4	39.0	3.6
2	2‐2	Yes	No	4.64	4.15	56.4	41.0	2.6
3	3‐1	Yes	Yes	5.01	4.42	62.8	34.5	2.6
3	3‐2	Yes	No	4.44	4.15	68.7	28.6	2.5
5	5‐1	No	Yes	5.73	5.15	62.7	34.7	2.6
5	5‐2	No	No	5.26	5.18	57.5	38.9	3.6
6	6‐1	No	Yes	5.25	5.34	56.9	39.5	3.5
6	6‐2	No	No	4.45	4.63	60.1	36.3	3.6
7	7‐1	No	Yes	4.56	4.35	54.3	43.1	2.6
7	7‐1	No	No	4.95	4.79	54.4	43.0	2.6
					Mean (%)	58.4	38.6	3.0
					SD (%)	4.4	4.3	0.5

### 3.6 Mineral Soil SWC Is Lower, but More Spatially Variable Under Rainfall Excluders

End‐of‐season local SWC spatial variability in the mineral soil was intensively characterized in the regular 6 × 7 plot grids yielding an overall mean and CV percentage for each plot (Table 3). We noted several trends: (1) mean SWC (= 15.57%) is about two times higher in plots with rainfall access (Exclusion: No) compared to those (SWC = 8.82%) where rain was diverted off the plots (Exclusion: Yes) (Pseudo‐*F*_1,10_ = 11.36, *p* = 0.0044). (2) Beech proliferation exerted no effect on moisture (*p* = 0.745), yet average moisture beneath excluders was 52% higher under profuse beech saplings (SWC = 10.57%) compared to beech‐free conditions (7.07%). (3) SWC spatial variability was two‐fold greater under rainfall excluders than in plots that were open to the atmosphere, that is, CV = 54.10% versus 24.40%, respectively (Exclusion: Pseudo‐*F*_1,8_ = 84.64, *p* = 0.0024).

**TABLE 3:** Volumetric soil water content (SWC) of the mineral soil (0–12 cm) that was measured in each plot grid using portable TDR on September 1, 2022; each estimate is the average of 42 readings. Spatial variation in moisture readings is expressed as a percentage coefficient of variation (CV%), Mean instantaneous decay rates (*k*‐values) are estimated from ln‐transformed litter mass‐remaining % (Day 90 only), averaged across leaf litter “species” and “meshcodes.”

Site	Plot	Beech	Exclude	Mean SWC (%)	SWC CV (%)	Instant‐*k* (year^−1^)
1	1‐1	Yes	Yes	12.6	50.75	−0.467
1	1‐2	Yes	No	22.81	22.87	−1.190
2	2‐1	Yes	Yes	9.71	48.62	−0.206
2	2‐2	Yes	No	14.20	22.55	−0.816
3	3‐1	Yes	Yes	9.40	53.13	—^a^
3	3‐2	Yes	No	11.26	29.29	−0.929
5	5‐1	No	Yes	5.98	66.08	−0.204
5	5‐2	No	No	12.87	24.34	−0.715
6	6‐1	No	Yes	8.43	46.66	−0.260
6	6‐2	No	No	18.59	26.24	−0.911
7	7‐1	No	Yes	6.79	59.36	−0.424
7	7‐2	No	No	13.71	21.10	−0.733

^a^ Due to incompleteness of Plot 3‐1 data entries for litter decay, no instantaneous *k*‐value was included as a preliminary estimate.

## 4 Discussion

From the results of our short‐term litter decay experiment, we confirmed several predictions, while refuting several others. As predicted, sugar maple leaf litter decayed more rapidly than did beech, given that the former is a higher quality substrate than the latter. However, we could not confirm that maple would decay more slowly in the presence of beech litter than in the absence of the latter; indeed, the opposite was true (see additivity tests below). Based on regression results, we further confirmed that the two‐species mixture decayed at rates intermediate between the monospecific litters, although responses of 50:50 mixtures tended to resemble maple decay rates (Figure 2). Based on the standardized differences and their associated error estimates, further tests (*Z*‐tests) would confirm that the mixtures behave in an additive rather than non‐additive manner, albeit over the very short term. Yet, guided by Ostrofsky's (2007) advice regarding tests that should include separate masses and separate decay rate estimates for the two (or more) species constituting mixed litter packs, we found that their respective maple and beech 90‐day instantaneous *k*‐values exceeded rates that were estimated from monospecific bags by 20%–58%. Such synergisms have been documented in the literature (Handa et al. 2014), possibly occurring through N‐transfer from one species to the other (Gartner and Cardon 2004; Schimel and Hättenschwiler 2007; Lummer et al. 2012). This mechanism may account for the observed 20% increase in mixed beech mass loss (Appendix 3). The 27% and 58% increases that were observed in mixed maple decay may have arisen from the accompanying beech litter tissues that were acting as a source of moisture (Collin et al. 2025) for microbial decomposers, despite also possibly facilitating the leaching of allelopathic or antimicrobial compounds from the beech (Hane et al. 2003). The antagonistic response in mixed beech (36% decrease) may be the result of N‐immobilization in maple litter, thereby slowing beech decay (Bonanomi et al. 2011).

At final removal (90 days), mixed litter lost just 2% more total mass in plots where beech proliferated than in beech‐free plots, while the difference in averaged (or expected) beech and maple mass losses between the same beech‐proliferated versus beech‐free plots was 5% (Table 4). Decay rates (Figure 2) would suggest that despite large 95% credible limits, differences between plots where beech saplings proliferated and beech‐free plots may be real, albeit subtle. Longer‐duration incubations of the litter bags (including an overwintering period) would reveal more concrete differences among treatments (Liu et al. 2020). Furthermore, rates of mass loss and final mass remaining would suggest that given the sensitivity of litter decay responses to soil moisture deficits, soils in which beech proliferated may provide a more favorable physical environment than does maple alone. Surface accumulations of organic material, especially beech leaf litter, possibly conserve more water in soils that are not covered with tents in the presence rather than in the absence of beech litter (Courcot et al. 2024).

**TABLE 4:** Final leaf mass remaining in the litter bag incubations. Mean (±SE) fractions at Day 90 are shown for Proliferation‐Exclusion‐Species combinations. Means in this column that are followed by a different letters are separated from one another by their respective upper and lower 95% confidence limits. Mass loss % estimated from mean remaining proportion; estimates in parentheses are individual species losses for mixed bags. Effects of mesh size (large, medium, small) on mean instantaneous *k*‐values are summarized in the last column on the right side of the table.

Prolif.	Exclude	Species	Mean‐remaining (SE)	95% LL	95% UL	Mass loss (%)	Mesh size L/M/S *k*‐values
Yes	Yes	Maple	0.929 c (0.019)	0.892	0.965	7.1 (9.2)	−0.264/−0.315/**−0.356**
Yes	Yes	Beech	0.924 c (0.019)	0.887	0.961	7.6 (7.3)	−0.315/**−0.365**/−0.315
Yes	Yes	Mixed	0.915 c (0.019)	0.899	0.972	8.5 (8.3)	**−0.852**/−0.416/−0.203
Yes	No	Maple	0.756 a (0.015)	0.743	0.803	24.4 (22.9)	−1.137/**−1.309**/−1.045
Yes	No	Beech	0.809 ab (0.015)	0.766	0.826	19.1 (20.9)	**−1.501**/−0.913/−0.903
Yes	No	Mixed	0.792 ab (0.015)	0.766	0.826	20.8 (21.9)	−0.690/**−1.248**/−0.994
No	Yes	Maple	0.930 c (0.015)	0.900	0.960	7.0 (8.8)	**−0.311**/−0.298/−0.291
No	Yes	Beech	0.927 c (0.015)	0.897	0.957	7.2 (4.9)	−0.315/−**0.365**/−0.284
No	Yes	Mixed	0.935 c (0.015)	0.908	0.967	6.5 (6.9)	−0.298/−0.237/−0.298
No	No	Maple	0.824 ab (0.015)	0.794	0.854	17.6 (22.4)	**−0.987**/−0.737/−0.717
No	No	Beech	0.845 b (0.015)	0.815	0.875	15.5 (16.3)	**−0.757**/−0.609/−0.690
No	No	Mixed	0.814 b (0.015)	0.784	0.844	18.6 (19.4)	−0.724/−0.839/**−1.014**

*Note:* Mean mass remaining at 90 days for 12 Proliferation‐Exclusion‐Species combinations. Mass loss % based on remaining proportions does not consistently differ from mass losses estimated from the individual species in mixed bags and shown in parentheses (Wilcoxon signed‐rank test: *Z* = −0.980). Ranking losses between these sets of estimates strongly agree (*r*_s_ = 0.786, *p* = 0.021). Under mixed bags, values in parentheses are averaged values of the aforementioned estimates. The final column includes instantaneous *k*‐values for L(arge), M(edium), and S(mall) mesh sizes, respectively. The fastest decay rate among the three mesh sizes is highlighted in boldface. No consistent ordering of decay rates could be objectively discerned among mesh sizes (Poor agreement: Kendall's *W* = 0.116, Chi‐square = 2.67, df = 2, *p* = 0.263).

According to differences in decay rates between beech‐sapling treatments, beech litter fall probably acts as a slowly decomposing blanket or mulch that retards surface soil moisture loss (Bélanger et al. 2019; Collin et al. 2025), and that spatial–temporal patterns of precipitation and moisture storage responses bear further scrutiny with respect to such forest floor variation (Yu et al. 2019). Indeed, this response suggests that forest floor variability and micro‐topography, together with altered physical or biochemical properties and microbial assemblages, are more subtly influencing decay rates (Hane et al. 2003; Midgley et al. 2015; Bélanger et al. 2019; Beugnon et al. 2023), warranting additional investigation. Mapping of the topography and vegetation at the stand‐ or landscape level using remote sensing tools, accompanied by extensive ground‐truthing, could permit future investigations to model forest tree composition and litter deposition and mixing, as described by Bigelow and Canham (2015), for example. Identifying micro‐sites on the forest floor could further be achieved by determining spatial patterns of tree species through various developmental stages, from seedling establishment to canopy dominance. In the context of our own forest inventories, our initial results suggest past disturbance within stands that now contain shade‐intolerant species in the canopy, and very likely a strongly delineated mosaic of micro‐sites.

Overall, these collective patterns could aid in identifying “hotspots” and “coldspots” of microbial decomposer activity and nutrient transformation across broader study areas; these “hot” or “cold” locations may be indicated by the differential deposition of beech litter. Alternatively, soil moisture measurements in the presence of both overstory and understory American beech in the plots may reflect how beech size‐age classes can exploit and partition moisture use over a growing season, go dormant, or reallocate water already stored in tissues (Köcher et al. 2013; Harrison et al. 2020; Cholet et al. 2022; Rasoanaivo et al. 2024). Thus, “spot” or “moment” identification is likely to be a more daunting task, given that their origin must be determined (e.g., rhizosphere, root mycobionts, aggregate surfaces) and the transient nature of “hot (and cold) moments” makes their monitoring difficult (Kuzyahov and Blagodatskaya 2015; Kooch et al. 2019). Thus, modest increases in variation that were explained by regression of decay rates including an additional covariate, such as moisture or pH, suggest either that inclusion of each plot‐level variable was an inappropriate choice or that they are estimates too crude or too few to reflect the actual temporal dynamics of edaphic properties, or spatial variation in forest floor composition, given that their estimated regression coefficients (*β*_13_) were not “significant” (i.e., most included zero in their credible intervals) and their inclusion did not improve *R* ^2^ by 5%, 10% or more. Despite their non‐zero credible intervals, the slope estimates (*β*_13_, *β*_14_, *β*_15_) for plot‐level variables that were collectively included in the multiple regression likewise had very small values, which did little to influence substantially the mass loss dynamics for any of the “species.”

Stresses such as drought have been experimentally integrated into litter bag incubation designs, as demonstrated in the present study and by many others (Knapp et al. 2024) for longer durations, including Quer et al. (2022) in Mediterranean forest. The latter authors examined the importance of environmental context and soil organisms on litter decay dynamics, together with the effects of drought on plant litter quality in terms of increased specialized metabolites and decreased litter nutrient contents. In situ incubation studies of litter cohorts can thus be made more useful in disentangling and identifying drivers among the many controlling factors and stress through the establishment of more complex long‐term inter‐site comparisons (e.g., across the nature reserve), reciprocal transplants (Krna et al. 2023), or common‐garden experiments (Prescott and Vesterdal 2021; Peng et al. 2022).

Indeed, longer periods of incubation, differences in drought exposure (magnitude, duration, extent and frequency; Knapp et al. 2024), forest floor manipulations (litter addition or removal; e.g., Prévost‐Bouré et al. 2010), winter snowpack augmentation or removal, and more detailed litter quality assessments would aid in addressing subsequent hypotheses, identifying common trends in mass loss trajectories, and formulating more comprehensive mechanistic explanations of decay processes. Under ongoing climate change, recurrent drought will not only affect existing plant growth and survival, but also the progress of predicted northward range shifts of canopy hardwood species.

Despite greater sapling recruitment of sugar maple, American beech, and red maple at the range limits of these northern hardwood species (Boisvert‐Marsh et al. 2019), sugar maple is more likely to be a casualty of poleward species migration into the boreal forest due to edaphic rather than climatic constraints, given that its specific seedbed and nutritional requirements may not be met by the lower quality of the boreal biome's soils (Collin et al. 2017, 2018; Carteron et al. 2020). While maple species other than *A. saccharum* have experienced “maple decline” (Boakye et al. 2023; Stern et al. 2023), red maple has seen an increase in abundance not only with its northern range recruitment, but also by its migration into the midwestern USA, along with American beech and sugar maple (Fei et al. 2017). The advance of this endomycorrhizal species into the boreal forest, rather than sugar maple, is likely through the former species establishment in canopy gaps (Leithead et al. 2010). In contrast, the continued persistence of sugar maple within its current range, together with the ecosystem services that it provides, would likely require targeted silvicultural prescriptions that diminish competition with American beech, while allowing entry of shade‐tolerant, early successional species into the canopy (Nelson and Wagner 2014; St‐Jean et al. 2021), and which would increase stand water yield (Gavinet et al. 2019). Forestry interventions that diminish drought effects could likely sustain high rates of litter decay leading to SOM formation, carbon sequestration, and soil nutrient‐pool replenishment.

Droughts that are quantified and qualified (i.e., duration, magnitude, frequency) through such monitoring are likely to affect nutrient mineralization and leaching, thereby prolonging Phase 1 decay. One simple decomposition predictor, that is, litter turnover with durations of 3–4 years rather than 1 year (Appendix 2), suggests that leaching arrested by drought would only be relieved in the overwintering period and spring snowmelt, if soil moisture deficiency were to continue into the autumn and later. Yet for how long, we cannot say unless soil moisture and temperature recording were to continue. These are premature speculations, given the brief exposure of the litter to field conditions driving their decay, and that predicting the progress of decomposition over the longer term assumes a more complicated and prolonged trajectory in the presence of intermittent and unanticipated moisture deficits.

In a comparable study, Jourdan and Hättenschwiler (2021) incubated leaf litter of European beech ( *Fagus sylvatica* L.), European silver fir ( *Abies alba* Mill.) and downy oak (*Quercus pubescens* Willd.) in monospecific litter bags, along with two‐species mixtures of beech combined with one of the other two species, under experimental drought conditions. The authors' imposition of prolonged summer soil‐moisture deficits resulted in slightly slower carbon loss (and presumably, organic matter) compared to controls, yet exerted no similar effects upon litter N. They further noted that decay of mixtures exhibited non‐additive effects on C and N release, giving credence to extending litter bag incubations well beyond the summer months. Further analyses of C and N concentrations in the present study of sugar maple and American beech could also confirm the previous authors' observations.

Forest litters typically converge upon a similar stable carbon composition (and element stoichiometry) in late stages of decomposition (Liu et al. 2020), which undermines the usefulness of litter traits (e.g., indices of initial litter quality, such as C:N, lignin:N, or leachable material) and short‐term decay studies in predicting the long‐term fate of the dead plant materials (Moore et al. 2017). Indeed, maple and beech leaves in our study had not been incubated within the forest floor for a sufficiently long duration (i.e., beyond Phase 1; Prescott 2010; Prescott and Vesterdal 2021) to complete leaching and to discern divergence in mass loss and mass loss rates among treatments, with eventual convergence upon asymptotic values. The interaction between litter traits and the environment is not well known, as has been noted by Canessa et al. (2021) and others (Pérez‐Suárez et al. 2012; Moore et al. 2017). Moreover, spatial–temporal variability that accompanies these responses increases as decomposition progresses and as they diverge, but remains largely unexplained, especially in relation to initial trait values (Moore et al. 2017).

Very short‐term studies that are much less than 3–5 years duration (Prescott 2010), like our exclusion experiment, may be useful venues for exploring divergence in mass loss rates through the strategic deployment of many litter bags across a wide range of micro‐site types (Bélanger et al. 2019), while mapping spatial variation in forest floor compositions (Bigelow and Canham 2015). In such a comprehensive experiment, more frequent litter bag retrievals and tissue quality assessments would follow, together with intensive recording of fine‐scale site environmental conditions commensurate with the frequency of sampling. A range of initial measured attributes may then be generally reflected or predicted in these later responses, thereby explaining a greater proportion of variation in mass loss rates. Yet, experimental duration might not be of sufficient length to develop a robust and more complex description of decay such as the double exponential model (labile and recalcitrant compartments; Pérez‐Suárez et al. 2012) or more flexible and biologically realistic Weibull functions. Yet, differences would likely begin to emerge well beyond Phase 1 over the longer duration of bag incubation, that is, after at least 1 year in the field (including one overwintering period, subsequent spring snowmelt, and summer growing conditions). These responses would also require supporting information in the form of dynamic relationships within the litter element ensemble and among its proximate carbon fractions.

We had predicted that moisture deficits would affect decomposition rates under rainfall excluders (Exclusion) more strongly than any of the other factors, including Proliferation, Species, and Meshcode. Even with free access to rainfall, we found no strong effects for either Species or Meshcode. With respect to the latter, we had predicted mass loss would increase with increasing litter bag aperture size, yet we observed equivocal results. Mass loss trends between large‐, medium‐, and fine‐mesh bags, which we had observed especially in the rain‐accessible plots, were not significant. Trends were very slight and non‐significant under rainfall exclusion, consistent with expectation. Indeed, no consistent ordering of decay rates could be objectively discerned among mesh sizes (Table 4). Thus, our initial prediction regarding mesh effects on mass loss could not be supported.

The soil macro‐fauna may be the agents that are most responsible for these changes, which can consequently affect fragmentation and subsequent physical processes, such as spillage. While the invasion of eastern North American hardwood forest by Eurasian earthworms can result not only in faster rates of litter disappearance, it can also exert potentially negative effects on soil physical structure, carbon, and water storage, and the diversity of the forest flora and fauna, which have not co‐evolved with these alien ecosystem engineers. Few to no large earthworms (mega‐fauna) were seen in the stands (C. Ghotsa Mekontchou, personal observations); forest stand invasion by lumbricids is typically manifested by the disappearance of the forest floor layer, understory herbs, and seedling recruits, and in altered soil structure. We did not observe these features of earthworm invasion, but their absence does not preclude the presence and activity of smaller annelid species in the faunal decomposer community that gained entry to the bags. Yet, the absence of earthworm invasions in these preserve sites affords further opportunities for drought and decomposition research that includes forest floor manipulations, with litter fall additions, removals, and substitutions with other single or multiple canopy species inputs.

American beech is a native species, and the only member of the genus *Fagus* in the Nearctic (1 of 12 species globally; Tubbs and Houston 1990). Yet, its proliferation in sugar maple stands is slowly replacing the latter species in the understory, thereby altering the composition and quality of the forest floor, perhaps adversely so (Hane et al. 2003). We do not know whether the 50%:50% mass ratio of bagged litter (i.e., litter cohorts) reflects the bulk litter falling onto the forest floor or the microflora experiencing this transition, or whether effects of beech litter inputs on decay and nutrient release would be diminished by increasing the maple:beech ratio, for example, to 75%:25% or 90%:10%. Moreover, these manipulations could continue to modify faunal and microbial activity and microbial community dynamics (Peng et al. 2022). There are precedents for such manipulations in litter cohort studies in field decomposition (Fyles and Fyles 1993; Gartner and Cardon 2004).

In the case of our study, litter bags with a maple:beech mass ratio of 25%:75% or lower (10%:90%) could be similarly included in further experiments to further test additivity effects and to illustrate this gradient of litter mixing at both local and broader scales within the Kenauk Preserve. Indeed, the gradient approach has involved systematic sampling along a transect from a one forest stand type to an adjacent different forest type in terms of edaphic properties, soil microbiota, bulk litter fall mass and composition, and biogeochemical processes and decomposition (Laganière et al. 2010). Along such a transect, Jacob et al. (2010) found that litter bag mixtures of higher quality litters, together with monospecific bags of these native European species ( *Acer pseudoplatanus* L., *A. platanoides* L., *Tilia cordata* Mill., *T. platyphyllos* Scop., *Carpinus betulus* L., *Fraxinus excelsior* L.), decomposed more rapidly in the field when moving along a gradient of decreasing European beech ( *Fagus sylvatica* L.) in the canopy and its litter accumulations on the forest floor in old‐growth stands in Germany.

Our experiment was conducted at the northern limits of eastern North American hardwood forest, yet its broadleaf overstory is reminiscent of Central European forest studied by Jacob et al. (2010) with the latter's three endomycorrhizal species (two *Acer* spp., one *Fraxinus*) and four ectomycorrhizal species (*Tilia* spp., *Carpinus* and *Fagus*). The authors found that the behavior of leaf mixture decay among pairs of these species (in the proportions that they were found in the forest plots) was additive along a gradient of decreasing *F. sylvatica* litter fall. Their canopy species are shade tolerant, with the exception of sycamore maple ( *A. pseudoplatanus* ). The composition of our own overstory includes shade‐intolerant red maple. Together with black cherry, its presence suggests that previous disturbance events had transpired, which were severe enough to disrupt existing understory seedling and sapling recruitment. This process would allow many more moderately to strongly shade‐intolerant species to fill canopy gaps that were created, such as *O. virginiana* , *P. serotina* , and *P. grandidentata* that are native to the region. Indeed, scenarios beyond typical stand‐level canopy gap dynamics could include extensive canopy removal through widespread decline and premature tree death in the case of sugar maple (Boakye et al. 2023) or intensive disease outbreaks (e.g., beech bark disease; Kish et al. 2022) leading to the loss of several to numerous beech trees from the overstory.

Early phase leaching of decaying sugar maple and American beech leaf litter, and their 50:50 mixture, was curtailed by the soil moisture deficiencies that were imposed on northern hardwood forest stands for two consecutive summers. In the face of ongoing climate change, the decrease in precipitation and litter decay rates interrupts leaching of the three aforementioned “species,” thereby forestalling the progress of leaf conversion to SOM and soil nutrient pool replenishment. The presence of recurring drought and competition from American beech threatens not only the long‐term persistence and continued vitality of sugar maple trees but also the range of ecosystem services that this species supplies, including biogeochemical processes such as nutrient mineralization. Under rainfall‐accessible conditions, ironically, beech litter covering the ground generates leachates that are allelopathic to maple seedling establishment while helping the soil to retain moisture, potentially increasing litter decay rates of the three species.

## 5 Conclusions

The 90‐day‐long mesh‐bag incubation study demonstrated that leaf litter mass loss rates of sugar maple, American beech, and their 50:50 mixtures were very sensitive to experimentally induced soil‐moisture deficits. Litter decay under rainfall excluders was about three times slower than decay that was not subject to moisture limitation. Rainfall exclusion not only reduced mean soil volumetric water content but also increased its spatial variability. Moisture likely was the principal driver of decomposition, while differences in litter quality (high‐quality maple vs. low‐quality beech) and litter bag mesh size exerted more subtle effects. Covariates that were related to soil properties and vegetation responses, which we incorporated into our litter decay equation, were gathered at the stand‐ and plot‐level, but these lacked sufficient spatial–temporal resolution to be useful predictors in the model. To better understand litter decay dynamics and the role of drought, the sufficiency or insufficiency of soil moisture stores should be monitored long‐term across many maple‐dominated stands with different forest floor conditions, litter fall production, and forest management regimes. This range of decomposition responses could provide more comprehensive data with which to generalize mass loss trajectories of litter fall in the northernmost regions of the hardwood forest.

## Author Contributions

**William F. J. Parsons:** formal analysis (lead), writing – original draft (lead). **Claudele Ghotsa Mekontchou:** conceptualization (equal), investigation (lead), methodology (lead). **Audrey Maheu:** conceptualization (equal), funding acquisition (lead), methodology (supporting), project administration (lead), supervision (equal), writing – review and editing (equal). **David Rivest:** conceptualization (equal), funding acquisition (supporting), methodology (supporting), supervision (equal), writing – review and editing (equal).

## Conflicts of Interest

The authors declare no conflicts of interest.

## Data Availability

The mass‐loss data that support the findings of this study are openly available at: https://doi.org/10.5281/zenodo.13991332.

## Appendix 1

Linear‐mixed model summary of litter decay responses to fixed effects of rainfall exclusion, litter bag mesh size, leaf litter “species” and beech proliferation based on instantaneous‐*k* estimates for each sample.

**Table:**

Source	df	*F*	*p*
Exclusion (Ex)	1, 501	326.251	< 0.001
MeshCode (MC)	2, 501	5.102	0.006
Species (S)	2, 501	3.898	0.021
Proliferation (Pr)	1, 3	0.247	0.654
Exclusion × MeshCode	2, 501	2.412	0.091
Exclusion × Species	2, 501	3.532	0.030
MeshCode × Species	4, 501	0.313	0.869
Exclusion × Proliferation	1, 501	3.824	0.051
MeshCode × Proliferation	2, 501	0.993	0.371
Species × Proliferation	2, 501	1.303	0.273
Exclusion × MeshCode × Species	4, 501	0.831	0.506
Exclusion × MeshCode × Pr	2, 501	1.138	0.321
Exclusion × Species × Pr	2, 501	0.637	0.529
MeshCode × Species × Pr	4, 501	0.387	0.818
Ex × MC × S × Pr	4, 501	0.269	0.898

*Note:* “Site” is used as a random effects grouping factor. df are the numerator and denominator degrees‐of‐freedom for each *F*‐statistic.

## Appendix 2

Bayesian linear regression model of ln‐percent mass‐remaining as a function of litter species composition, rainfall exclusion (Yes vs. No) and beech proliferation (Yes vs. No) in the understory and incubation duration (year‐fractions). Different letters beside *k*‐values indicate where 95% Credible Intervals (Lower, Upper) around the *k*‐value estimates do not overlap.

**Table:**

Proliferation	Exclude	Spp.	Pred. *k*_*i*_	*k*‐value (year^−1^)	SD		Lower CI	Upper CI	Mixed bag *k*	% Change
—	—	—	*β*_0_	4.508	0.007		4.503	4.513	—	—
Yes	Yes	Maple	*β*_1_	−0.248	0.065	d	−0.367	−0.115	−0.393	58.5
Yes	Yes	Beech	*β*_2_	−0.286	0.065	d	−0.407	−0.155	−0.308	7.7
Yes	Yes	Mixed	*β*_3_	−0.323	0.065	d	−0.444	−0.192	—	—
Yes	No	Maple	*β*_4_	−1.026	0.065	a	−1.147	−0.895	−1.058	3.1
Yes	No	Beech	*β*_5_	−0.787	0.065	bc	−0.907	−0.656	−0.950	20.7
Yes	No	Mixed	*β*_6_	−0.969	0.065	a	−1.089	−0.838	—	—
No	Yes	Maple	*β*_7_	−0.327	0.056	d	−0.433	−0.213	−0.378	15.6
No	Yes	Beech	*β*_8_	−0.307	0.056	d	−0.412	−0.193	−0.207	−32.6
No	Yes	Mixed	*β*_9_	−0.289	0.056	d	−0.395	−0.175	—	—
No	No	Maple	*β*_10_	−0.812	0.056	b	−0.918	−0.698	−1.031	27.0
No	No	Beech	*β*_11_	−0.698	0.056	c	−0.803	−0.583	−0.727	4.1
No	No	Mixed	*β*_12_	−0.829	0.056	b	−0.935	−0.715	—	—

*Note:* The predictor designations and their associated slope coefficients (*β*_*i*_) in the GLM are numbered according to the associated treatment combinations referred to in the equation in the text (Methods: Statistical analysis). The back‐transformed intercept value (*β*_0_) is 90.1%. The 95% credible limits [LC, UC] are shown for each estimate. The last two columns include individual mass loss rates (instantaneous‐*k*) and percentage change for decaying maple and beech leaves that were retrieved from mixed litter bags at 90 days. Estimates in boldface fall beyond the adjacent credible intervals that were estimated for slope parameters in the multiple regression equation.

## Appendix 3

Basal area (m^2^ ha^−1^) contributions of nine canopy tree species in the 12 experimental plots, Kenauk Nature Reserve (Quebec). Endomycorrhizal (AM) or ectomycorrhizal (ECM) status (Mycorr.) of the roots of each species is indicated above each four‐letter species acronym (see Table 1 for the Latin binomials). Overall basal area means and standard deviations (SD) are summarized at the bottom of the table. According to PERMANOVA (univariate responses), *p*‐values indicate canopy species BA differences with and without beech sapling proliferation.

**Table:**

Site	Proliferation	Mycorr.	AM	ECM	AM	AM	ECM	ECM	AM	ECM	ECM
		Exclude	ACSA	FAGR	ACRU	ACPE	OSVI	BEAL	PRSE	TIAM	POGR
1	Yes	Yes	20.43	7.52	7.34	0.00	0.00	0.17	0.00	0.00	0.00
1	Yes	No	9.65	8.85	9.41	0.85	0.00	6.51	1.17	0.00	0.00
2	Yes	Yes	27.16	0.73	0.00	0.94	0.08	0.00	0.25	0.00	0.75
2	Yes	No	25.25	5.47	0.00	3.22	0.38	0.14	0.89	0.00	5.87
3	Yes	Yes	18.23	3.40	0.00	0.07	0.29	0.00	3.03	0.00	0.00
3	Yes	No	19.38	5.94	0.00	0.12	0.00	3.79	1.8	0.00	0.00
5	No	Yes	26.37	0.00	0.00	0.00	0.83	0.00	0.00	7.60	0.00
5	No	No	38.35	0.00	0.00	0.05	3.63	0.00	0.00	2.05	0.00
6	No	Yes	30.25	0.00	0.00	0.00	0.00	0.00	0.00	0.00	0.00
6	No	No	32.46	0.00	0.00	0.02	0.13	0.00	0.00	0.00	0.00
7	No	Yes	33.40	0.00	0.00	0.00	3.13	0.00	0.00	0.00	0.00
7	No	No	27.16	0.01	0.00	0.01	4.16	0.00	0.00	0.00	0.00
		Mean BA	25.67	2.66	1.40	0.44	1.05	0.88	0.60	0.80	0.55
		SD BA	7.82	3.40	3.29	0.94	1.59	2.08	0.97	2.22	1.69
		*p*	**0.008**	**< 0.002**	—	0.196	**0.039**	—	—	0.453	—

*Note:* Species, from most (1) to least shade‐tolerant (4): ACSA, *Acer saccharum* (1); FAGR, *Fagus grandifolia* (1); ACPE, *A. pensylvanicum* (1); OSVI, *Ostrya virginiana* (2); ACRU, *A. rubrum* (3); TIAM, *Tilia americana* (3); BEAL, *Betula alleghaniensis* (3); PRSE, *Prunus serotina* (3); POGR, *Populus grandidentata* (4). Rare species (not included): TSCA, *Tsuga canadensis* (1); FRAM, *Fraxinus americana* (3). *p*‐values in bold type indicate significant differences in canopy species BA between proliferation treatments.

## Appendix 4

Soil water content (SWC, %) that was measured using portable TDR on September 1, 2022 on 12 plots that were established on sugar maple‐dominated hardwood forest stands (Kenauk Reserve, Quebec). Readings were taken at 42 points across 6 rows (uppercase letters) and 7 columns (numbers) of a grid that was superimposed on each 20 m × 20 m plot, with roughly three‐meter spacing between points. The value at each grid node that is depicted in the graph is the mean of three consecutive readings taken using 12 cm wave‐guides that were inserted vertically into the surface mineral soil (means are summarized in the accompanying table). Exemplar data that have been presented here are for Site 1‐1 (plot covered by a rainfall excluder tent during the summers of 2021 and 2022; see Figure 1 and Table 3).
